# Chinese genetic variation database of inborn errors of metabolism: a systematic review of published variants in 13 genes

**DOI:** 10.1186/s13023-023-02726-1

**Published:** 2023-06-12

**Authors:** Yongchao Guo, Jianhui Jiang, Zhongyao Xu

**Affiliations:** 1 Shenzhen Uni-medica Technology Co., Ltd, Liuxian Culture Park, Nanshan District, 5180553 Shenzhen, China; 2 Maternal and Child Health Hospital of Guangdong Province, No.13 Guangyuan West Road, Yuexiu District, 510010 Guangzhou, Guangdong Province China

**Keywords:** Population-specific variant database, Chinese, Inborn errors of metabolism, Variant interpretation

## Abstract

**Background:**

Population-specific variation database of inborn errors of metabolism (IEMs) is essential for precise genetic diagnosis and disease prevention. Here we presented a systematic review of clinically relevant variants of 13 IEMs genes reported among Chinese patients.

**Methods:**

A systematic search of the following electronic databases for 13 IEMs genes was conducted: PubMed-NCBI, China national knowledge infrastructure and Wanfang databases. Patient data was extracted from articles eligible for inclusion and recorded in Excel electronic form using a case-by-case approach.

**Results:**

A total of 218 articles, 93 published in English and 125 in Chinese, were retrieved. After variant annotation and deduplication, 575 unique patients (241 from articles published in Chinese) were included in the population-specific variation database. Patients identified by newborn screening and symptomatic presentation were 231 (40.17%) and 344 (59.83%), respectively. Biallelic variants were observed in 525/575 (91.3%). Among the 581 unique variants identified, 83 (14.28%) were described ≥ 3 times and 97 (16.69%) were not recorded in Clinvar or HGMD. Four variants were reclassified as benign and dozens of confusing variants deserved further research.

**Conclusion:**

This review provides a unique resource of the well-characterized diseases and causative variants that have accumulated in Chinese population and is a preliminary attempt to build the Chinese genetic variation database of IEMs.

**Supplementary Information:**

The online version contains supplementary material available at 10.1186/s13023-023-02726-1.

## Introduction

Inborn errors of metabolism (IEMs) are a group of inherited disorders caused by variants in genes coding for proteins that function in metabolism. As part of the newborn screening (NBS) programs, more than 40 IEMs can be screened by tandem mass spectrometry (MS-MS). According to a national cross-sectional survey, these IEMs occur in 1 in 2585 births in China [1].

IEMs are usually diagnosed through a combination of enzyme activity analysis, clinical findings and molecular analysis. A key component in molecular analysis is how to interpret variants accurately. Standardized assertion criteria to classify variants associated with Mendelian disorders were developed by the American College of Medical in 2015 [2]. And then the Clinical Genome Resource (ClinGen) Inborn Errors of Metabolism Working Group created a comprehensive, standardized knowledge base of genes and variants for metabolic diseases [3]. For a given variant, a comprehensive review of reported patients carrying the variant is essential for pathogenicity determination. For example, if a variant in a gene for a recessive disorder is in trans with a known pathogenic variant, this can be considered moderate evidence for pathogenicity (PM3). And this evidence could be upgraded to strong or very strong if there are multiple observations (observed in multiple patients).

Aside from genetic diagnosis, next-generation sequencing (NGS) based newborn genetic screening and carrier screening of common diseases, including IEMs, have been widely explored [4–7]. These programs commonly require a comprehensive knowledge base of clinically relevant variants, especially those discovered in patients from a certain population [8].

As variant frequencies vary considerably among different populations, building population-specific variation database is essential for precise genetic diagnosis, newborn genetic screening and carrier screening [9–11]. PubMed-NCBI and the Human Gene Mutation Database (HGMD) are commonly used for searching relevant publications and variants in concern, but the information is scattered and those published in non-English journals could not be retrieved or with only English abstracts available.

Here we present a systematic review of clinically relevant variants of 13 IEMs genes reported among Chinese patients through searching public databases in English and also in Chinese. This work is a preliminary attempt to develop the Chinese genetic variation database of IEMs.

## Materials and methods

### Search strategy

A systematic search of the following electronic databases for 13 IEMs genes was conducted: PubMed-NCBI, China national knowledge infrastructure (CNKI) and Wanfang databases. These genes include *ACADM* (medium-chain acyl-CoA dehydrogenase deficiency, MCADD), *ACADVL* (very long-chain acyl-CoA dehydrogenase deficiency, VLCADD), *ACAT1* (beta-ketothiolase deficiency, BKD), *ASS1* (classic citrullinemia, CTLN1), *BCKDHA/BCKDHB* (maple syrup urine disease, MSUD), *BTD* (biotinidase deficiency, BTDD), *CPS1* (carbamoylphosphate synthetase I deficiency, CPSID), *GCDH* (glutaricaciduria, type I, GAI), *HLCS* (holocarboxylase synthetase deficiency, HLCSD), *IVD* (isovaleric acidemia, IVA) and *PCCA/PCCB* (propionic acidemia, PA). The search strategy was structured using a combination of terms including “gene symbol or disease name” and “China, Chinese, Taiwan or Hong Kong” in PubMed database, “gene symbol or disease name” in CNKI and Wanfang databases. Variants and relevant publications referring to Chinese patients in the Human Gene Variant Database (HGMD® Professional 2021.4) were also collected. Two reviewers independently screened titles and abstracts of all articles published by the end of December 2021 to determine if articles were suitable for inclusion. Publications with full-text available and detailed clinical or biochemical and genotype information of individual patients were included. Those involving prenatally diagnosed patients and dissertations were excluded.

### Data extraction and variant annotation

The following patient data was extracted from articles eligible for inclusion and recorded in Excel electronic form using a case-by-case approach: genetic variants, mode of identification (newborn screening or symptomatic presentation), zygosity and pedigree analysis.

Extracted variants were annotated according to the guidelines of the Human Genome Variation Society (HGVS) nomenclature. Validation of variant annotations was performed by Name Checker (https://mutalyzer.nl/name-checker) for exonic variants and by Variant Validator (https://variantvalidator.org/) for intronic variants. Patients with unverifiable variants were recorded and excluded.

Deduplication.

For patients with identical genotype, clinical characteristics, the research group and affiliations were checked to allow the exclusion of those reported later. Siblings were counted as a unique patient. After deduplication, all unique patients were enrolled in the further analysis.

## Result

### Overview and summary statistics

**Fig. 1:**
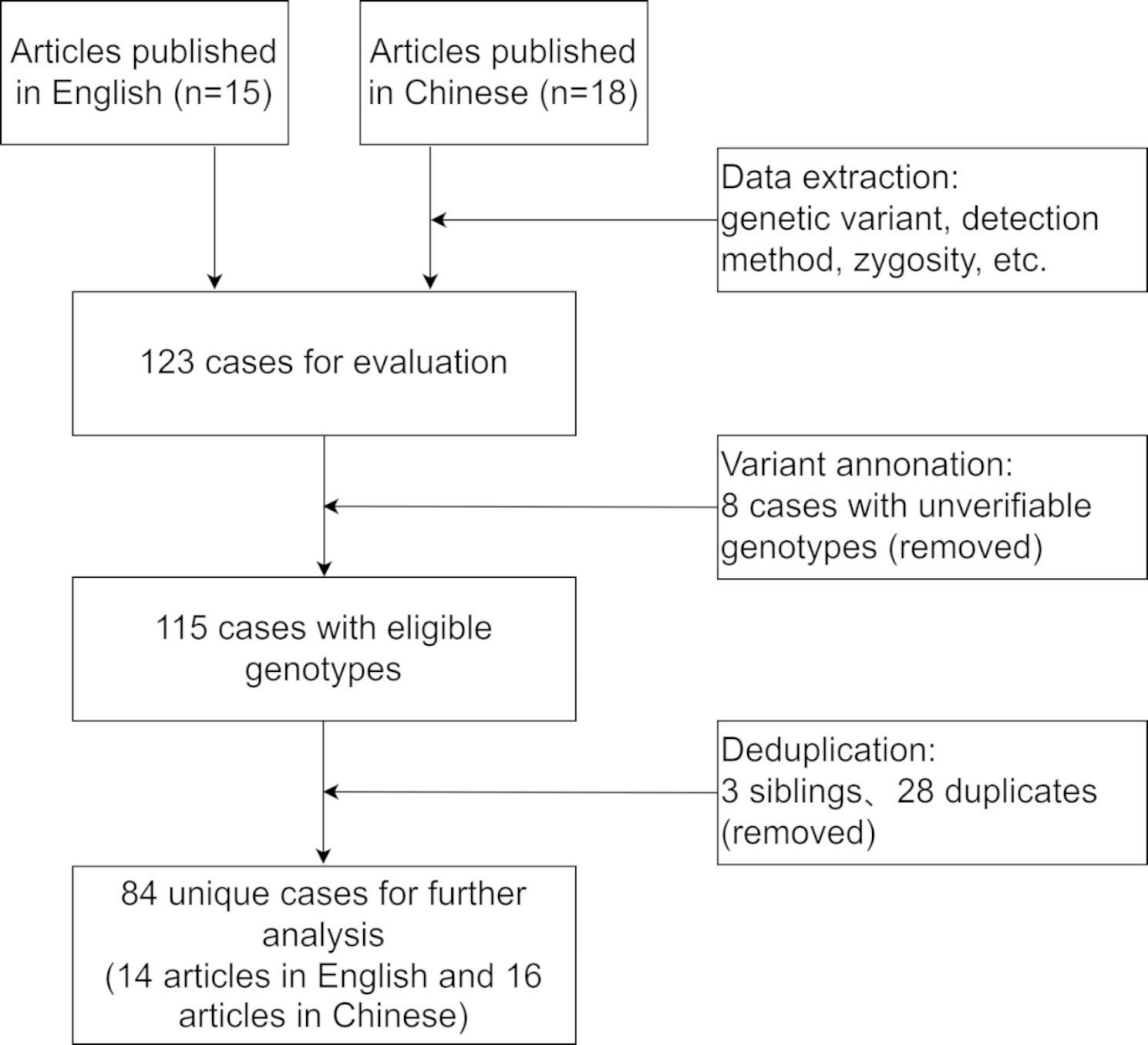
Flow chart of the review strategy used in this study. Take *ACADVL* as an example

Flow chart of the review strategy using *ACADVL* gene as an example was shown in Fig. 1. Briefly, 15 articles in English and 18 in Chinese were identified through database search. After data extraction, 123 patients confirmed with VLCADD were collected. Eight cases with unverifiable genotype information, three siblings, and twenty-eight duplicates were removed after variant annotation and deduplication. This ultimately resulted in 84 unique patients referring to 14 English and 16 Chinese articles.

**Fig. 2:**
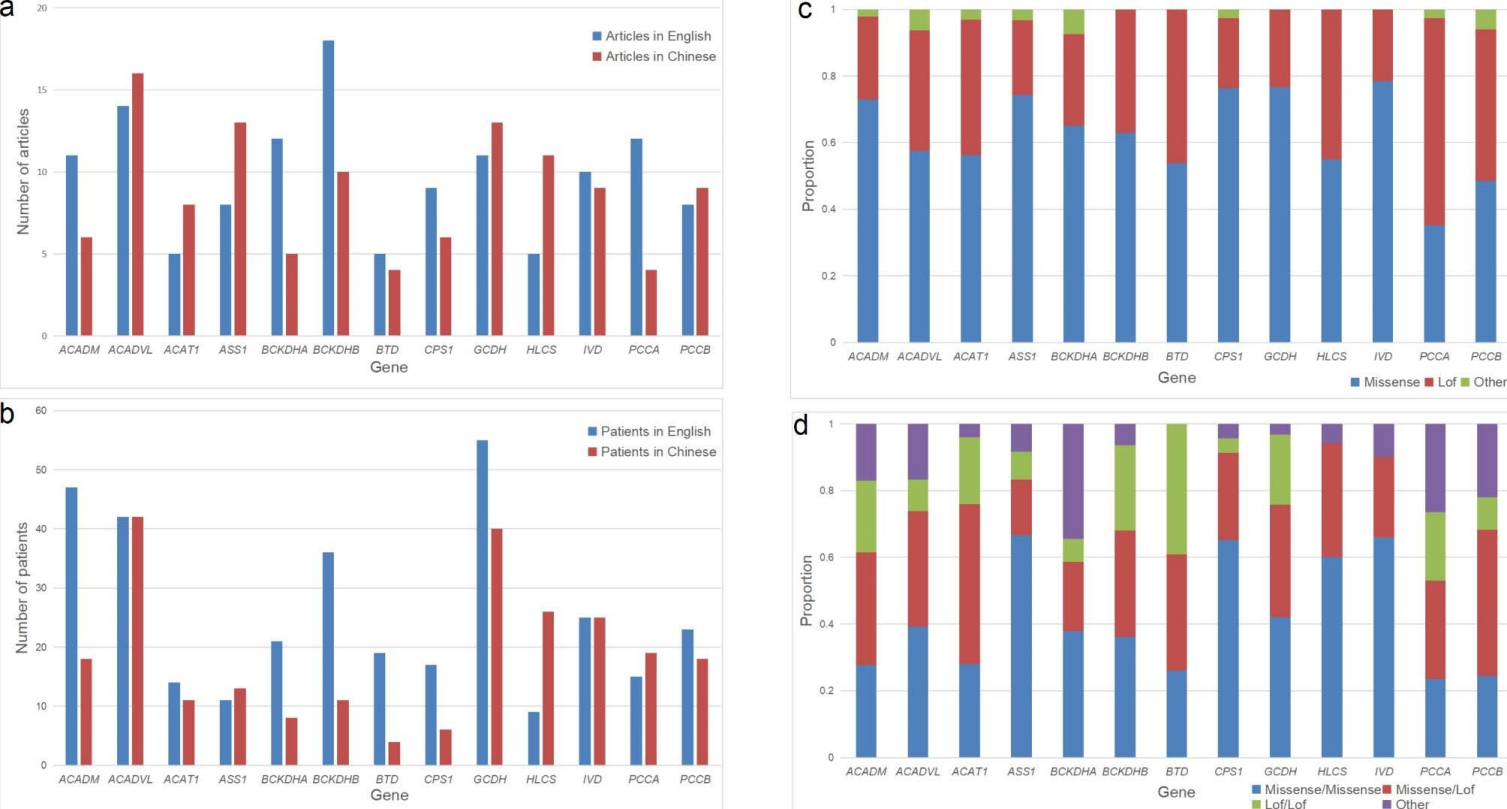
Distribution of articles and unique patients reviewed, proportion of variant effect and combination for each gene. **a** Number of articles published in English and Chinese. **b** Number of patients published in English and Chinese articles. **c** Proportion of missense and loss-of-function (Lof) variants. Other: Proportion of in-frame insertion/deletions. **d** Proportion of patients with missense/missense, missense/Lof and Lof/Lof. Other: Proportion of patients with in-fame insertion/deletions and only one allele identified

Taking all genes together, 218 articles (93 in English and 125 in Chinese) were retrieved through database search. Of the 736 patients collected, 161 did not meet the inclusion criteria and were removed, resulting in 575 unique patients (in 91 English and 106 Chinese articles), of which 241 (41.91%) were from articles published in Chinese. The detailed information and inclusion/exclusion reasons on a case-by-case basis were listed in Additional file 1 (Table S1). The number of unique patients and articles reviewed for each gene were shown in Fig. 2a and b.

The patients identified by NBS and symptomatic presentation were 231 (40.17%) and 344 (59.83%), respectively (Table 1). Significant deviation from the general level was observed in several IEMs. Most of MCADD patients were identified by newborn screening (87.69%), while most of MSUD, BTDD, CPSID and HLCSD patients were identified by symptomatic presentation (86.96%~96.55%). Biallelic variants were observed in 525/575 (91.3%) patients, including 105/575 (18.26%) in homozygous and 420/575 (73.04%) in compound heterozygous. Hence, the clinical diagnosis was not genetically confirmed in 8.70% of cases (heterozygous). This proportion was especially high for MCADD, MSUD (*BCKDHA*) and PA (*PCCA*) (15.38%, 24.14% and 23.53%, respectively).

**Table 1:** Basic information of the 575 unique IEMs patients identified in Chinese population

IEMs (Gene)	Mode of identification			Zygosity		
	Newborn screening, n (%)	Symptomatic presentation, n (%)		Homozygous, n (%)	Compound heterozygous, n (%)	Heterozygous, n (%)
MCADD *(ACADM)*	57 (87.69)	8 (12.31)		15 (23.08)	40 (61.54)	10 (15.38)
VLCADD *(ACADVL)*	51 (60.71)	33 (39.29)		4 (4.76)	74 (88.10)	6 (7.14)
BKD *(ACAT1)*	11 (44.00)	14 (56.00)		5 (20.00)	20 (80.00)	0 (0.00)
CTLN1 *(ASS1)*	11 (45.83)	13 (54.17)		4 (16.67)	19 (79.17)	1 (4.17)
MSUD *(BCKDHA)*	1 (3.45)	28 (96.55)		4 (13.79)	18 (62.07)	7 (24.14)
MSUD *(BCKDHB)*	5 (10.64)	42 (89.36)		10 (21.28)	34 (72.34)	3 (6.38)
BTDD *(BTD)*	3 (13.04)	20 (86.96)		5 (21.74)	18 (78.26)	0 (0.00)
CPSID *(CPS1)*	1 (4.35)	22 (95.65)		1 (4.35)	22 (95.65)	0 (0.00)
GAI *(GCDH)*	32 (33.68)	63 (66.32)		24 (25.26)	68 (71.58)	3 (3.16)
HLCSD *(HLCS)*	2 (5.71)	33 (94.29)		14 (40.00)	19 (54.29)	2 (5.71)
IVA *(IVD)*	29 (58.00)	21 (42.00)		4 (8.00)	41 (82.00)	5 (10.00)
PA *(PCCA)*	11 (32.35)	23 (67.65)		4 (11.76)	22 (64.71)	8 (23.53)
PA *(PCCB)*	17 (41.46)	24 (58.54)		11 (26.83)	25 (60.98)	5 (12.19)
Total	231 (40.17)	344 (59.83)		105 (18.26)	420 (73.04)	50 (8.70)

Abbreviations: IEMs, inborn errors of metabolism; MCADD, medium-chain acyl-CoA dehydrogenase deficiency; VLCADD, very long-chain acyl-CoA dehydrogenase deficiency; BKD, beta-ketothiolase deficiency; CTLN1, classic citrullinemia; MSUD, maple syrup urine disease; BTDD, biotinidase deficiency; CPSID, carbamoylphosphate synthetase I deficiency; GAI, glutaricaciduria, type I; HLCSD, holocarboxylase synthetase deficiency; IVA, isovaleric acidemia; PA, propionic acidemia

Of note, four variants, i.e., *ACADVL* c.128G > A, *BCKDHA* c.452 C > T, *HLCS* c.126G > T and *PCCA* 1850T > C, which were erroneously reported as disease-causing variants, were reclassified as benign according to Clinvar (multiple submitters, no conflicts). These variant were detected in 7 cases, including 1 with VLCADD (VLCADD-020), 1 with MSUD (MSUDIa-025), 2 with HLCSD (HLCSD-011 and HLCSD-012), and 3 with PA (PA-A001, PA-A003 and PA-A020) (Additional file 1: Table S1).

### Variant spectrum

All the unique variants identified were listed in Additional file 2 (Table S2) and Table 2 was a short version including those with allele number ≥ 3 (recurring variants).

**Table 2:** Unique variant statistics and recurring variants of the 13 IEMs genes

Gene	Unique Variants	Variants recorded in Clinvar/HGMD (%)	Recurring variants (allele count ≥ 3)		
			Nucleotide Alteration	Predicted Effect on Protein	Allele Count (%)
*ACADM*	48	43 (89.58)	c.449_452del	p.(Thr150ArgfsTer4)	37 (28.46)
			c.1085G > A	p.(Gly362Glu)	10 (7.69)
			c.387 + 1del	-	6 (4.62)
			c.580 A > G	p.(Asn194Asp)	6 (4.62)
			c.157 C > T	p.(Arg53Cys)	3 (2.31)
			c.727 C > T	p.(Arg243Ter)	3 (2.31)
			c.985 A > G	p.(Lys329Glu)	3 (2.31)
			c.1040G > T	p.(Gly347Val)	3 (2.31)
*ACADVL*	94	67 (71.28)	c.1349G > A	p.(Arg450His)	21 (12.50)
			c.553G > A	p.(Gly185Ser)	7 (4.17)
			c.664G > C	p.(Gly222Arg)	5 (2.98)
			c.1280G > A	p.(Trp427Ter)	5 (2.98)
			c.1532G > A	p.(Arg511Gln)	5 (2.98)
			c.65 C > A	p.(Ser22Ter)	4 (2.38)
			c.298_299del	p.(Gln100ValfsTer3)	4 (2.38)
			c.1077 + 6T > A	-	3 (1.79)
			c.1276G > A	p.(Ala426Thr)	3 (1.79)
			c.1396G > T	p.(Asp466Tyr)	3 (1.79)
			c.1505T > A	p.(Leu502Gln)	3 (1.79)
*ACAT1*	32	29 (90.63)	c.622 C > T	p.(Arg208Ter)	5 (10.00)
			c.121-3 C > G	-	4 (8.00)
			c.1006-1G > C	-	4 (8.00)
			c.1124 A > G	p.(Asn375Ser)	4 (8.00)
			c.997G > C	p.(Ala333Pro)	3 (6.00)
*ASS1*	31	26 (83.87)	c.1087 C > T	p.(Arg363Trp)	5 (10.42)
			c.380G > A	p.(Arg127Gln)	3 (6.25)
			c.431 C > G	p.(Pro144Arg)	3 (6.25)
			c.1168G > A	p.(Gly390Arg)	3 (6.25)
*BCKDHA*	40	34 (85.00)	c.117dup	p.(Arg40GlnfsTer11)	3 (5.17)
			c.647 C > T	p.(Ala216Val)	3 (5.17)
*BCKDHB*	54	46 (85.19)	c.331 C > T	p.(Arg111Ter)	5 (5.32)
			c.550del	p.(Ser184ProfsTer46)	5 (5.32)
			c.853 C > T	p.(Arg285Ter)	5 (5.32)
			c.93_103dup	p.(Phe35TrpfsTer41)	3 (3.19)
			c.508 C > T	p.(Arg170Cys)	3 (3.19)
			c.523T > C	p.(Phe175Leu)	3 (3.19)
			c.659del	p.(Gln220ArgfsTer10)	3 (3.19)
			c.1028del	p.(Ser343LeufsTer9)	3 (3.19)
*BTD*	26	25 (96.15)	c.1433dup	p.(Leu478PhefsTer13)	6 (13.04)
			c.577del	p.(His193ThrfsTer51)	5 (10.87)
			c.1324del	p.(Arg442GlyfsTer39)	4 (8.70)
			c.175 C > T	p.(Arg59Cys)	3 (6.52)
			c.1190_1191delinsAG	p.(Val397Glu)	3 (6.52)
*CPS1*	38	35 (92.11)	c.1145 C > T	p.(Pro382Leu)	4 (8.70)
			c.3443T > A	p.(Met1148Lys)	3 (6.52)
*GCDH*	77	72 (93.51)	c.1244-2 A > C	-	51 (26.84)
			c.532G > A	p.(Gly178Arg)	7 (3.68)
			c.1064G > A	p.(Arg355His)	5 (2.63)
			c.533G > A	p.(Gly178Glu)	5 (2.63)
			c.148T > C	p.(Trp50Arg)	4 (2.11)
			c.406G > T	p.(Gly136Cys)	4 (2.11)
			c.892G > A	p.(Ala298Thr)	4 (2.11)
			c.1147 C > T	p.(Arg383Cys)	4 (2.11)
			c.1204 C > T	p.(Arg402Trp)	4 (2.11)
			c.1207 C > T	p.(His403Tyr)	4 (2.11)
			c.1261G > A	p.(Ala421Thr)	4 (2.11)
			c.109_110del	p.(Gln37GlufsTer5)	3 (1.58)
			c.263G > A	p.(Arg88His)	3 (1.58)
			c.395G > A	p.(Arg132Gln)	3 (1.58)
			c.413G > A	p.(Arg138Lys)	3 (1.58)
			c.416 C > G	p.(Ser139Trp)	3 (1.58)
			c.1205G > A	p.(Arg402Gln)	3 (1.58)
*HLCS*	20	15 (75.00)	c.1522 C > T	p.(Arg508Trp)	31 (45.59)
			c.1088T > A	p.(Val363Asp)	10 (14.71)
			c.782del	p.(Gly261ValfsTer20)	3 (4.41)
			c.1544G > A	p.(Ser515Asn)	3 (4.41)
*IVD*	51	36 (70.79)	c.1199 A > G	p.(Tyr400Cys)	17 (17.00)
			c.149G > A	p.(Arg50His)	8 (8.00)
			c.205G > A	p.(Asp69Asn)	4 (4.00)
			c.467G > C	p.(Gly156Ala)	4 (4.00)
			c.631 A > G	p.(Thr211Ala)	4 (4.00)
			c.350G > A	p.(Arg117Gln)	3 (3.00)
			c.1186G > C	p.(Asp396His)	3 (3.00)
*PCCA*	37	31 (83.78)	c.2002G > A	p.(Gly668Arg)	12 (17.65)
			c.229 C > T	p.(Arg77Trp)	6 (8.82)
			c.1288 C > T	p.(Arg430Ter)	4 (5.88)
			c.1118T > A	p.(Met373Lys)	3 (4.41)
*PCCB*	33	25 (75.76)	c.1301 C > T	p.(Ala434Val)	11 (13.41)
			c.838dup	p.(Leu280ProfsTer11)	10 (12.2)
			c.1087T > C	p.(Ser363Pro)	7 (8.54)
			c.167_179delinsC	p.(Asp56_Lys60delinsAla)	6 (7.32)
			c.1316 A > G	p.(Tyr439Cys)	4 (4.88)
			c.-4156_184-1585del	-	3 (3.66)

Overall, 581 unique variants were identified in 575 unique patients. Twenty (3.44%) were large insertion/deletions distributed in nine genes, ninety-six (16.52%) were small insertion/deletions and others were single nucleotide variants. Missense and loss-of-function (Lof) variants (nonsense, frameshift, canonical +/−1 or 2 splice sites and other predicted Lof variants) accounted for 64.03% and 33.22%, respectively. Others were in-frame insertion/deletions. For *PCCA*, more than half were Lof variants (62.16%) (Fig. 2c). Patients with a missense variant on both alleles predominated in 8 genes, especially for *ASS1* (66.67%), *CPS1* (65.22%), *HLCS* (60.00%) and *IVD* (66.00%) (Fig. 2d). Patients with missense/Lof accounted for the highest proportion in *ACADM*, *ACAT1*, *PCCA* and *PCCB*, while those with Lof/Lof predominated only in *BTD*. By compared with public database, 97 variants (16.69%) were not recorded in Clinvar and HGMD. The proportion ranged from 29.21% for *IVD* to 3.85% for *BTD.*

Four hundred variants (68.85%) were detected only once. Eighty-three recurring variants were observed, with a maximum of 17 in *GCDH* and a minimum of 2 in *BCKDHA* and *CPS1*. The combined allele frequency of recurring variants was ≥ 50% in *ACADM, GCDH, HLCS* and *PCCB*, and < 20% in *BCKDHA* and *CPS1.*

There were 13 variants with an allele frequency ≥ 10%, including c.449_452del [p.(Thr150ArgfsTer4)] (28.46%) in *ACADM*, c.1349G > A [p.(Arg450His)] (12.50%) in *ACADVL*, c.622 C > T [p.(Arg208Ter)] (10.00%) in *ACAT1*, c.1087 C > T [p.(Arg363Trp)] (10.42%) in *ASS1*, c.1433dup [p.(Leu478PhefsTer13)] (13.04%) and c.577del [p.(His193ThrfsTer51)] (10.87%) in *BTD*, c.1244-2 A > C (26.84%) in *GCDH*, c.1522 C > T [p.(Arg508Trp)] (45.59%) and c.1088T > A [p.(Val363Asp)] (14.71%) in *HLCS*, c.1199 A > G [p.(Tyr400Cys)] (17.00%) in *IVD*, c.2002G > A [p.(Gly668Arg)] (17.65%) in *PCCA*, and c.1301 C > T [p.(Ala434Val)] (13.41%) and c.838dup [p.(Leu280ProfsTer11)] (12.20%) in *PCCB*.

### Variants with conflicting interpretations of pathogenicity

We searched the Genome Aggregation Database (gnomAD) to evaluate the allele frequencies of 581 unique variants in East Asian population. As expected, the allele frequency distribution observed for most of the variants identified in patients mirrored those in East Asian population (Additional file 2: Table S2). But as shown in Table 3, there were 30 variants (allele count ≤ 2) with an allele frequency in cases inconsistent with that in gnomAD when compared to recurring variants. These variants all had an allele frequency ≥ 0.0001 in gnomAD within range of recurring variants or even out of range. When referring to variant interpretation in Clinvar, 7 were labeled with conflicting interpretations of pathogenicity and 7 were labeled with variant of uncertain significance (VUS). We selected the *ACADVL* c.1434G > A variant as an example. This variant was detected in only one VLCADD patient, but had an allele frequency of 0.002105 in gnomAD, which was much higher than that of the most common allele c.1349G > A (0.0002506). And in Clinvar, 3 out of 4 submitters interpreted it as VUS.

**Table 3:** Variants (allele count ≤ 2) with allele frequency in cases inconsistent with that in gnomAD compared to recurring variants

Gene	Nucleotide Alteration	Predicted Effect on Protein	Interpretation in Clinvar*	Allele Count in cases (%)	Frequency in gnomAD (East Asian)	Frequency range of recurring variants in gnomAD (East Asian)
*ACADM*	c.668T > C	p.(Ile223Thr)	PAT(1)/VUS(1)	2 (1.54)	0.0001087	0.00005437–0.0002719
	c.617G > A	p.(Arg206His)	PAT(2)/LP(1)	1 (0.77)	0.0001631	
*ACADVL*	c.878 + 1G > C	-	PAT(1)	2 (1.19)	0.0001088	0.00005437–0.0002506
	c.1405 C > T	p.(Arg469Trp)	PAT(6)/LP(1)	2 (1.19)	0.0001087	
	c.863T > G	p.(Phe288Cys)	-	1 (0.6)	0.0001087	
	c.1226 C > T	p.(Thr409Met)	PAT(1)/VUS(2)	1 (0.6)	0.0003508	
	c.1434G > A	p.(Met478Ile)	PAT(1)/VUS(3)	1 (0.6)	0.002105	
*ACAT1*	c.83_84del	p.(Tyr28CysfsTer38)	PAT(3)	1 (2.00)	0.0001034	0.00005437–0.0002114
	c.163T > A	p.(Phe55Ile)	-	1 (2.00)	0.0001002	
*ASS1*	c.470G > A	p.(Arg157His)	PAT(3)/VUS(1)	1 (2.08)	0.0001002	0
	c.1004G > A	p.(Arg335His)	VUS(1)	1 (2.08)	0.0001002	
*BCKDHB*	c.818 C > T	p.(Thr273Ile)	-	2 (2.13)	0.0002179	0-0.0000544
	c.1159 C > T	p.(Arg387Ter)	PAT(3)/VUS(1)	1 (1.06)	0.0003806	
*BTD*	c.1246G > A	p.(Glu416Lys)	LP(1)/VUS(3)	2 (4.35)	0.000451	0-0.0001087
*CPS1*	c.2407 C > G	p.(Arg803Gly)	PAT(1)	2 (4.35)	0.0004018	0.00005025
	c.3538G >A	p.(Ala1180Thr)	VUS(2)	1 (2.17)	0.0003262	
	c.3793 C > T	p.(Pro1265Ser)	-	1 (2.17)	0.000641	
	c.4088_4099del	p.(Leu1363_Ile1366del)	LP(1)	1 (2.17)	0.0001002	
*GCDH*	c.1063 C > T	p.(Arg355Cys)	PAT(2)/LP(2)	2 (1.05)	0.0002007	0-0.001303
	c.300G > A	p.(Met100Ile)	VUS(1)	1 (0.53)	0.0002175	
	c.873 C > A	p.(Asn291Lys)	VUS(3)	1 (0.53)	0.000641	
	c.938G > A	p.(Arg313Gln)	PAT(1)/LP(1)	1 (0.53)	0.0001004	
	c.1156 C > G	p.(Arg386Gly)	PAT(1)/LP(1)	1 (0.53)	0.0002175	
*HLCS*	c.2010-1G > A	-	VUS(1)	1 (1.47)	0.0001093	0.0001002–0.0002718
*IVD*	c.457-2 A > G	-	LP(1)	2 (2.00)	0.0001087	0.00005437–0.0002175
	c.233G > A	p.(Arg78Gln)	-	1 (1.00)	0.0003262	
	c.539 C > T	p.(Ala180Val)	VUS(1)	1 (1.00)	0.0001002	
	c.823G > C	p.(Val275Leu)	VUS(1)	1 (1.00)	0.000451	
*PCCA*	c.688 C > T	p.(Arg230Cys)	LP(1)/VUS(1)	1 (1.47)	0.0001003	0-0.0001087
	c.1353 + 5_1353 + 9del	-	PAT(2)	1 (1.47)	0.0001089	

Abbreviations: PAT, pathogenic; LP, likely pathogenic; VUS, variant of uncertain significance. *Arabic numerals in the parentheses indicated the number of submitters

In addition, the allele frequency of a recurring variant in *HLCS*, c.782del [p.(Gly261ValfsTer20)], was similar to that of c.1522 C > T [p.(Arg508Trp)] (0.0002507 vs. 0.0002718) in gnomAD but was significantly different in Chinese patients (4.41% vs. 45.59%). Similar pattern was also observed for *IVD* c.631 A > G [p.(Thr211Ala)] when compared to c.1199 A > G [p.(Tyr400Cys)], i.e., 0.0002175 vs. 0.0002506 in gnomAD and 4.00% vs. 17.00% in patients.

As shown in Table 4, the *GCDH* c.1261G > A [p.(Ala421Thr)] variant was detected in 4 Chinese GAI patients, namely GAI-054, GAI-091, GAI-093, and GAI-094, all combined with c.1244-2 A > C (homozygous in GAI-054). GAI-054, GAI-093, and GAI-094 were identified by NBS [12, 13], while GAI-091 were identified by a clinical exome sequencing cohort for developmental disorders [14]. Pedigree analysis was clear only in GAI-091 (c.1244-2 A > C from father and c.1261G > A from mother). The c.1261G > A variant is classified as pathogenic/likely pathogenic by most of the Clinvar submitters. Six more GAI patients carrying this variant from Malaysia (Malaysian Chinese) [15], Europe [16] and America [17] were identified by further literature search, but none carried the c.1244-2 A > C variant simultaneously. This raised the possibility that there was strong linkage disequilibrium between c.1261G > A and c.1244-2 A > C in Chinese population.

**Table 4:** Summary of glutaricaciduria type I patients carrying the GCDH c.1261G > A variant reported in literature

Patient ID	Genotype	Race	Reference
GAI-054	c.1244-2 A > C/c.1244-2 A > C/c.1261G > A	Chinese	This review
GAI-091	c.1244-2 A > C/c.1261G > A	Chinese	This review
GAI-093	c.1244-2 A > C/c.1261G > A	Chinese	This review
GAI-094	c.1244-2 A > C/c.1261G > A	Chinese	This review
Patient 3	c.1063 C > T/c.1261G > A	Chinese (in Malaysia)	Abdul et al. (2016)
-	c.356 C > T/c.1261G > A	European	Christensen et al. (2004)
-	c.1204 C > G/c.1261G > A	European	Christensen et al. (2004)
Patient 3	c.1204 C > T/c.1261G > A	American?	Guenzel et al. (2021)
Patient 4	c.1204 C > T/c.1261G > A	American?	Guenzel et al. (2021)
Patient 5	c.1204 C > T/c.1261G > A	American?	Guenzel et al. (2021)

## Discussion

In this review, we collected clinically relevant variants of 13 IEMs genes reported among Chinese patients from articles published in English and Chinese till December 2021. Of the 575 unique patients identified, 241 (41.91%) were from Chinese articles, and of the 581 unique variants collected, 97 (16.70%) were not recorded in Clinvar or HGMD. This collection of up-to-date data into one database will reduce the labor of both researchers and clinicians, facilitate variant interpretation and provide variant spectrum information for newborn genetic screening and carrier screening. Besides, this review presents valuable and novel information as follows.

Relatively more patients with only one allele identified in MCADD, MSUD (*BCKDHA*) and PA (*PCCA*) (15.38%, 24.14% and 23.53%, respectively) highlight the fact that some variants are missed by the most common sequencing methods currently used, such as Sanger sequencing and exon-focused next-generation sequencing. These variants might involve large insertion/deletions, deep intronic variants or those located in the 5’ and 3’ untranslated regions. This was further demonstrated by a recent study that rare and recurrent variants located deep within *PAH* introns were not uncommon in phenylketonuria patients in China [18]. A concern should be raised that some variants located in exons detected in IEMs patients, especially those with high allele frequencies in population databases, might cover up the true disease-causing variants mentioned above.

The population frequency of a variant can be used as evidence both for and against pathogenicity. Based on common sense, a variant that is more often detected in cases should have a higher allele frequency in population databases. But genetic spectrum analysis in this review revealed that there were more than 30 variants with allele frequencies in cases inconsistent with those in gnomAD (East Asian). Several reasons are listed as follows. First, cases with well-characterized variants are less likely to be published and therefore the allele might be underrepresented [19]. Second, there are 56 ethnic groups in China and it is well known that the allele frequency differs considerably among ethnic groups. A previous study showed that the overall carrier frequency of 11 recessive diseases in China ranged from 4.15% in Hani ethnicity to 81.35% in Li ethnicity [20]. Therefore, allele frequency in IEMs cases collected from published articles might be skewed due to underrepresentation of some ethnic groups when taking Chinese as a whole. And also the allele frequency in gnomAD (East Asian) is not an effective data resource for Chinese population as some variants are only detected in Japanese or Korean population. The last but not least, some variants might be erroneously reported as disease-causing in literature and should be interpreted cautiously, especially those with allele frequencies higher than that of the most common variant. The finding highly suggests that the clinical significance of a variant for a specific population should be comprehensively evaluated.

Linkage disequilibrium was highly suspected between *GCDH* c.1261G > A and c.1244-2 A > C in Chinese population according to 4 cases with the c.1261G > A/c.1244-2 A > C genotype collected in this systemic review. Variant co-occurrence (phasing) information of c.1261G > A and c.1244-2 A > C in gnomAD (East Asian) might further support this inference. There are three individuals carrying the c.1261G > A variant, also carrying the c.1244-2 A > C variant simultaneously (Additional file 3: Figure. S1). As this paper was in preparation, another two cases with the same genotype identified by NBS were also reported in China [6]. As compound heterozygous status was only clear in one case, the degree of linkage disequilibrium between these two variants should be further discussed and pedigree analysis must be strictly performed for definite diagnosis. The latter could also be verified indirectly using the Integrative Genomics Viewer (IGV) during next-generation sequencing as c.1261G > A was close to c.1244-2 A > C. The exact pathophysiology leading to abnormal NBS results for newborns with this haplotype also deserves further research, which might be similar to asymptomatic *ASS1* carriers with high blood citrulline levels [21, 22].

This systematic review is a preliminary attempt to build the Chinese genetic variation database of IEMs. Next, we will gradually improve the database by covering more and more IEMs. As published cases and variants might represent only a small proportion of all, multilateral cooperation is imperative to enrich the database through sharing unpublished data. And for more common IEMs with hundreds or thousands of patients reported, such as phenylketonuria and methylmalonic aciduria, the cooperation is especially important for accurate profiling of unique cases. A web-based visual database is essential for data sharing, maintenance and regular updating of newly reported cases and variants, which is already in preparation.

In conclusion, this systematic review provides a unique resource of the well-characterized IEMs and causative variants that have accumulated in Chinese population, which is essential for precise genetic diagnosis and disease prevention.

**Abbreviations**.

IEMs: Inborn errors of metabolism; NBS: Newborn screening; MS-MS: Tandem mass spectrometry; NGS: Next-generation sequencing; HGMD: Human Gene Mutation Database; CNKI: China national knowledge infrastructure; MCADD: Medium-chain acyl-CoA dehydrogenase deficiency; VLCADD: Very long-chain acyl-CoA dehydrogenase deficiency; BKD: Beta-ketothiolase deficiency; CTLN1: Classic citrullinemia; MSUD: Maple syrup urine disease; BTDD: Biotinidase deficiency; CPSID: Carbamoylphosphate synthetase I deficiency; GAI: Glutaricaciduria, type I; HLCSD: Holocarboxylase synthetase deficiency; IVA: Isovaleric acidemia; PA: Propionic acidemia; HGVS: Human Genome Variation Society; VUS: Variant of uncertain significance; PAT: Pathogenic; LP: Likely pathogenic.

## Electronic supplementary material

Below is the link to the electronic supplementary material.

Supplementary Material 1

Supplementary Material 2

Supplementary Material 3

## Data Availability

All data generated or analysed during this study are included in this published article [and its supplementary information files].
