# Supplementary material for: Chinese genetic variation database of inborn errors of metabolism: a systematic review of published variants in 13 genes
Source: Orphanet J Rare Dis. 2023 Jun 12;18:148. doi: 10.1186/s13023-023-02726-1 (PMC10262587; doi:10.1186/s13023-023-02726-1)
Supplement: Supplementary file 3 — Supplementary Material 3 [file 13023_2023_2726_MOESM3_ESM.docx]

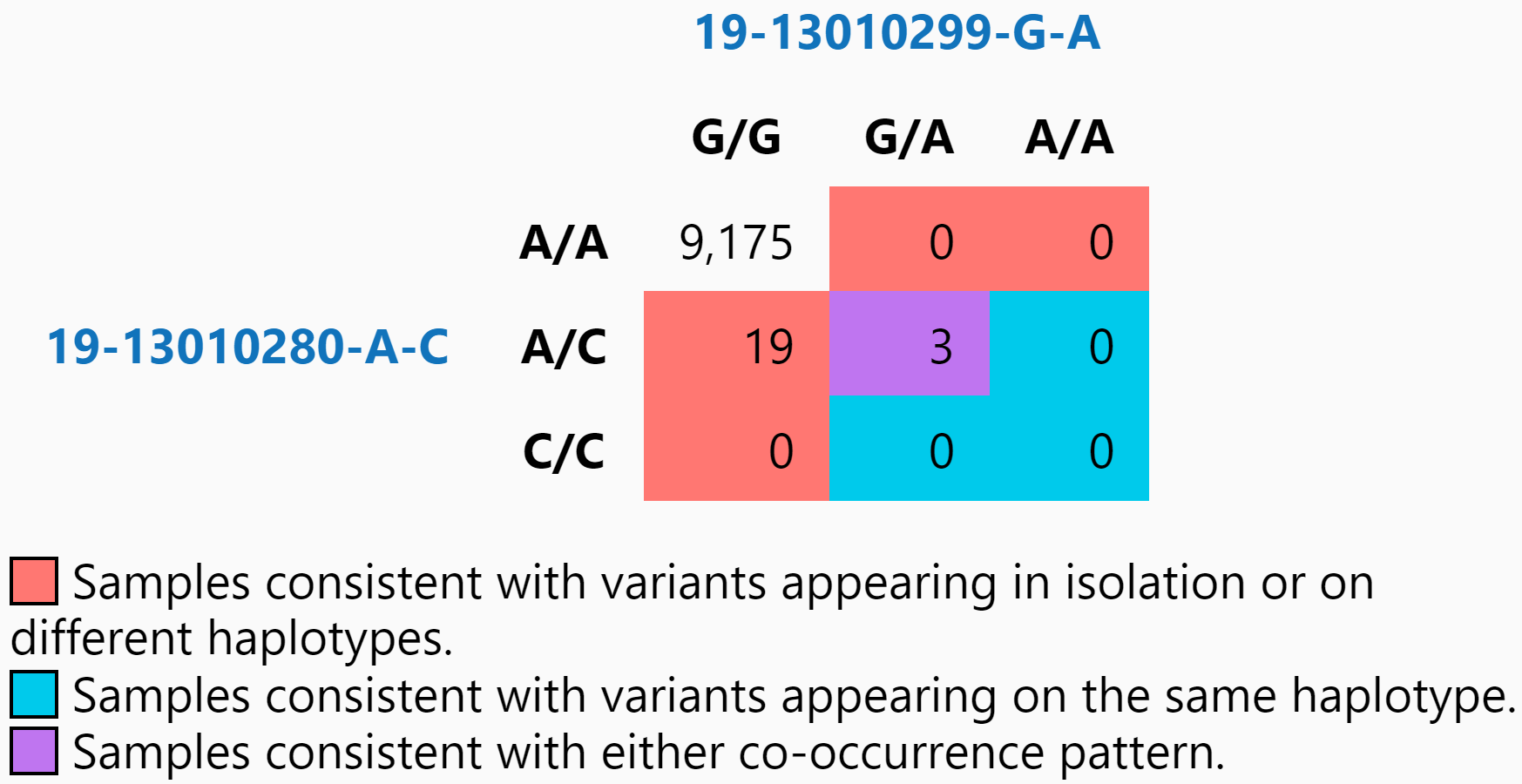


**Figure. S1** Variant co-occurrence (phasing) information of *GCDH* c.1261G>A and c.1244-2A>C in gnomAD (East Asian). 19-13010299-G-A:c.1261G>A; 19-13010280-A-C: c.1244-2A>C.
